# Supplementary material for: Systematic Investigations of Annealing and Functionalization of Carbon Nanotube Yarns
Source: Molecules. 2020 Mar 4;25(5):1144. doi: 10.3390/molecules25051144 (PMC7179235; doi:10.3390/molecules25051144)
Supplement: Supplementary file 1 [file molecules-25-01144-s001.pdf]

molecules

Supporting Information

Systematic investigations of annealing and functionalization of carbon nanotube yarns

Maik Scholz [1,2], Yasuhiko Hayashi[3*], Victoria Eckert[1,2], Vyacheslav Khavrus[1§], Albrecht Leonhardt[1], Bernd Büchner[1,4], Michael Mertig[2,5], Silke Hampel[1*]

[1] Leibniz Institute for Solid State and Material Research Dresden, Helmholtzstr. 20, 01069 Dresden, Germany
[2] Institute for Physical Chemistry, Technical University of Dresden, 01062 Dresden, Germany
[3] Graduate School of Natural Science and Technology, Okayama University, 3-1-1 Tsushima-naka, Kita, Okayama 700-8530, Japan
[4] Institute for Solid State Physics, Technical University of Dresden, 01062 Dresden, Germany
[5] Kurt-Schwabe-Institut für Mess- und Sensortechnik e.V. Meinsberg, 04736 Waldheim, Germany
[§] Current address: Life Science Inkubator Sachsen GmbH & Co. KG, Tatzberg 47, 01307 Dresden, Germany (Vyacheslav Khavrus)

* Correspondence: E-mail: S.Hampel@ifw-dresden.de; Tel: +49 (0) 351-4659-323 (S.H.) and E-mail: hayashi.yasuhiko@okayama-u.ac.jp; Tel: +81 (0) 86-251-8230 (Y.H.)

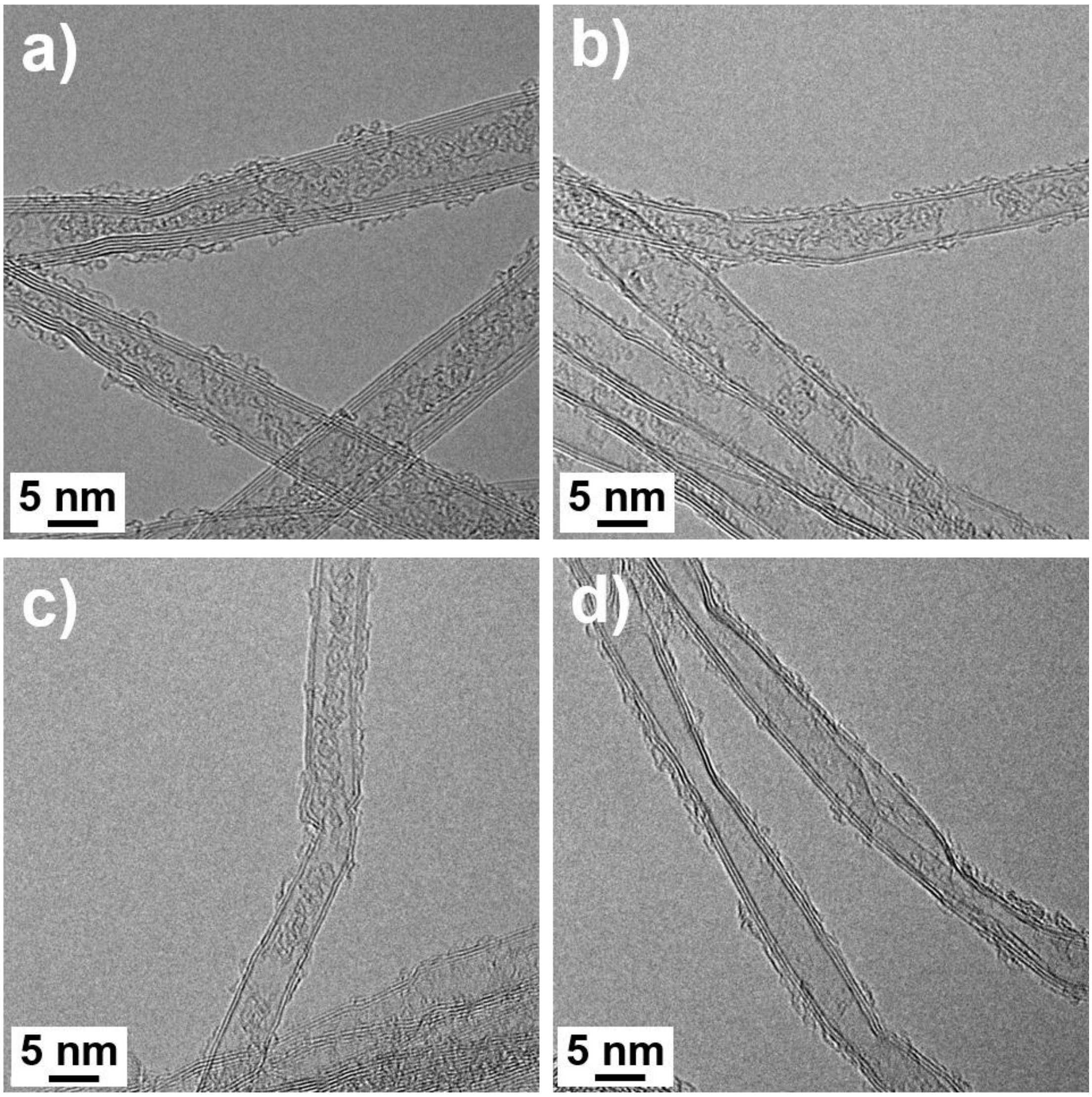

Figure S1: TEM images of pristine CNY.

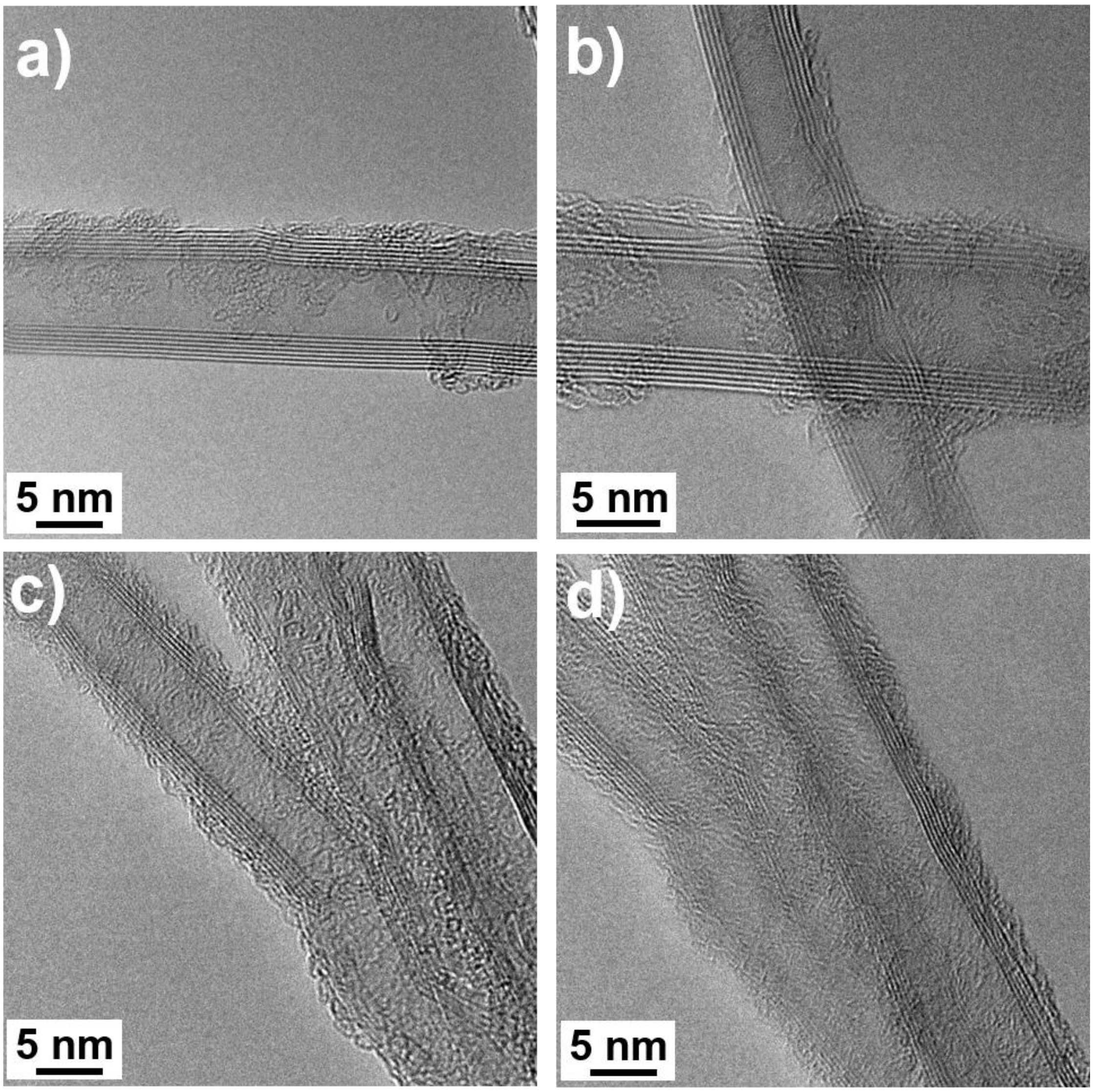

Figure S2: TEM images of annealed CNY for 2h at 2500°C

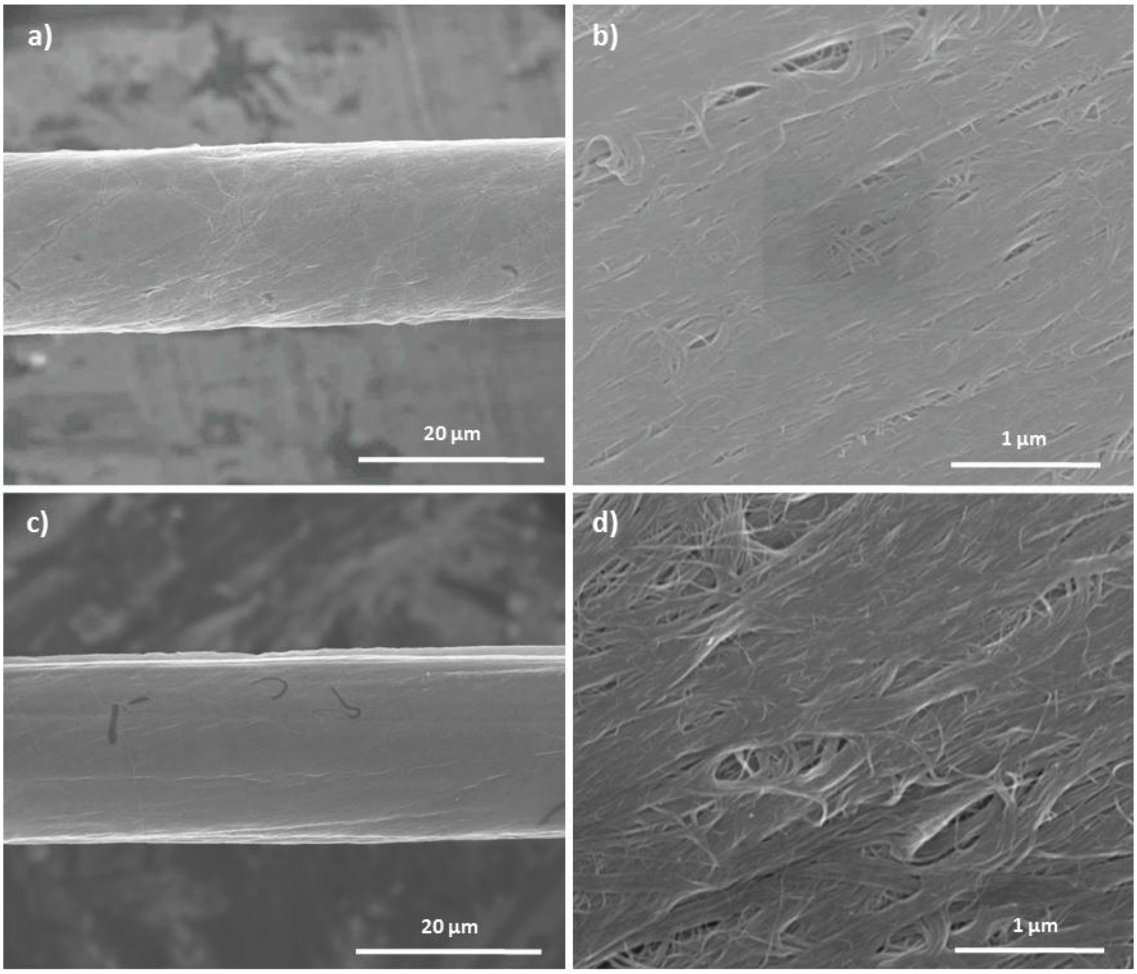

Figure S3: SEM images of CNY after treatment with H_2O_2: a) and b) after 3 h and c) and d) after 216 h treatment time.

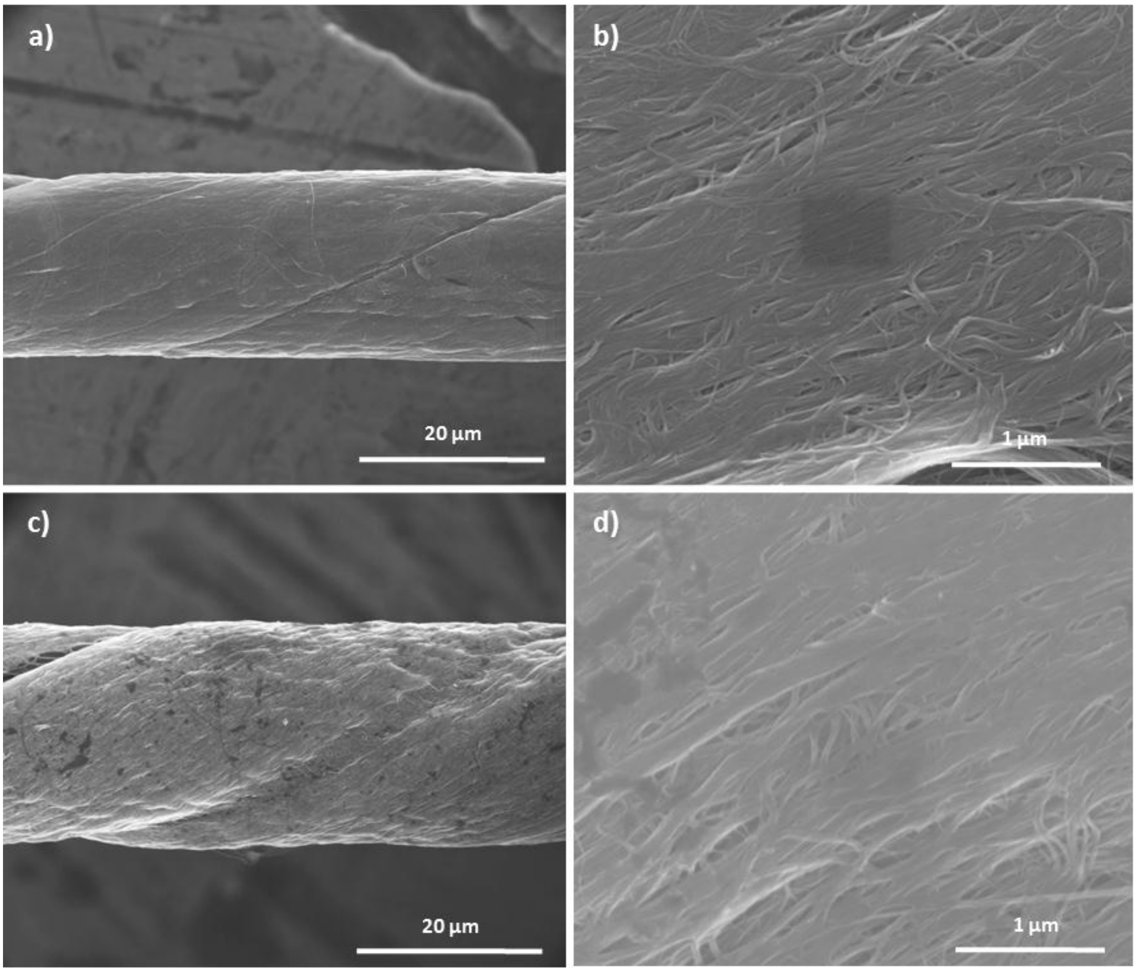

Figure S4: SEM images of CNY after treatment with HCl: a) and b) after 3 h and c) and d) after 216 h treatment time.

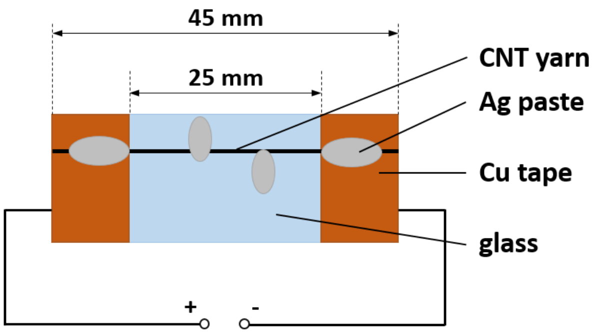

Figure S5: Detailed schematic illustration of the sample holder with fixed CNY for four-point measurements.
